# Tubo-ovarian mass with raised CA-125 in a 21-year-old female

**DOI:** 10.1186/s12957-022-02651-w

**Published:** 2022-06-08

**Authors:** Roli Purwar, Kishan Soni, Ragini Tilak, Ashish Verma, Manoj Pandey

**Affiliations:** 1 Department of Surgical Oncology, Institute of Medical Sciences, Banaras Hindu University, Varanasi, 221005 India; 2 Department of Microbiology, Institute of Medical Sciences, Banaras Hindu University, Varanasi, 221005 India; 3 Department of Radiodiagnosis and Imaging, Institute of Medical Sciences, Banaras Hindu University, Varanasi, 221005 India

**Keywords:** Peritonitis, Infection, Fungal, Curvularia, Young

## Abstract

**Introduction:**

Peritonitis associated with fungal species *Curvularia lunata* seldom occurs with only five cases reported in the literature, all in middle-age patients with comorbidities undergoing dialysis.

**Case report:**

A 21-year-old female who was referred to surgical oncology OPD with a diagnosis of ovarian malignancy, based on raised *cancer antigen 125* (CA 125) and suspected tubo-ovarian mass (TOM) on *magnetic resonance imaging* (MRI). A review of the MRI showed a pelvic collection with TOM, suggestive of infective pathology. Fungal culture and mass spectroscopy of the cystic collection identified the presence of *Curvularia lunata.* She was treated with oral itraconazole which showed symptomatic improvement and radiological response. In the follow-up period, the patient developed chest wall swelling, aspiration and geneXpert® revealed multidrug-resistant (MDR) tuberculosis, and treatment was started.

**Conclusions:**

Unusual causes of TOM and raised CA 125 should be kept in mind when dealing with young patients, as the possibility of epithelial ovarian cancer in this age is very low.

**Supplementary Information:**

The online version contains supplementary material available at 10.1186/s12957-022-02651-w.

## Introduction

Fungal peritonitis is an uncommon entity, which is associated with significant morbidity and mortality. Although most of the fungal peritonitis cases that have been reported are caused by *Candida albicans*, there are other rare fungal species that have also been identified [1]. *Curvularia lunata* is one such organism which seldom causes infection in humans. It is a saprobic dematiaceous fungus that is primarily found in the soil [2].

*C. lunata* was first described in 1898 by Wakker and Went, when they isolated it from dead sugar cane leaves. It was first suspected as a human pathogen in 1959, when Baylet isolated it from black grain mycetoma in Senegal [2]. A review of the literature revealed a handful of cases caused by *C. lunata* in humans, which includes mycetoma, paranasal sinusitis, peritonitis, cerebral phaeohyphomycosis, and disseminated disease infecting the eye, skin, pleura, lung, spine, brain, etc. In this article, we report a rare case of fungal peritonitis associated with *Curvularia lunata* in a female followed by multidrug-resistant (MDR) tuberculosis.

## Case

A 21-year-old sexually virgin female, from a middle-class family, was referred to Surgical Oncology outpatient in June 2020 with a provisional diagnosis of epithelial ovarian malignancy on the basis of magnetic resonance imaging (MRI) findings and raised cancer antigen 125 (CA125) level of 129.8 U/ml (normal 0–35). On enquiring, she had irregular menstruation for the last 6 months, abdominal distension for 2 months, and pain in the lower abdomen for the last 1 month. There was no history of fever or night sweats. She had a past history of cervical lymph node tuberculosis in 2017, for which she completed antitubercular therapy for 18 months with a standard regimen of isoniazid, rifampicin, pyrazinamide, and ethambutol. Other than this, there was no significant medical and surgical history. Personal and family history was noncontributory. On clinical examination, the general condition was fair and vitals were stable, neurological, respiratory, and cardiovascular systems, and were within normal limits. On per abdomen examination, the patient had mild tenderness in the lower abdomen with no signs of guarding, rigidity, or free fluid in the abdomen. No definite mass or lump was palpable.

**Fig. 1:**
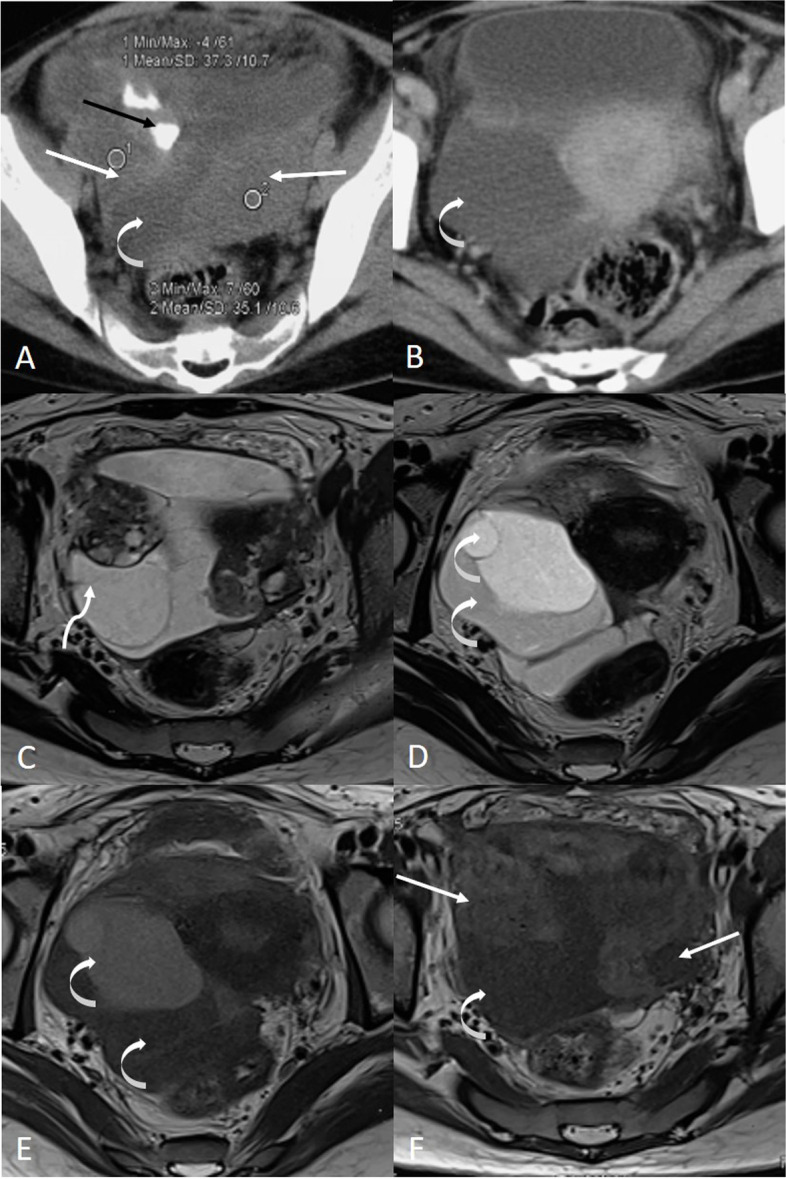
Axial MRI images of the pelvis before treatment showing the uterus in the center with a multiloculated cystic lesion being hypoattenuating on the CT scan (**A**, **B**), variably hyperintense on T2-weighted MRI (**C**, **D**), and variably hyperintense to hypointense on T1-weighted (**E**, **F**) images. The lesion also shows calcification on the CT scan (black straight arrow in **A**) and variable intensity pattern (curved arrows) suggesting that there are cystic components of various compositions. The lesion also shows a partial septa sign (twisted arrow in **C**), and this along with close circumferential relation with ovaries (straight white arrows in all sections) clearly indicated towards a possible tubo-ovarian origin of the mass. The bulk of both ovaries is however visualized normally (straight white arrows)

Her previous MRI scan was reviewed which showed extensive smooth peritoneal thickening with moderate ascites, multiloculated cystic lesion in bilateral adnexa, variably hyperintense on T2-weighted MRI, and variably hyperintense to hypointense on T1-weighted images, and the lesions were in close relation with ovaries but bilateral ovaries were normally visualized (Fig. 1). This suggested a possibility of tubo-ovarian origin of the mass. Her routine biochemical blood investigations (complete blood count, renal and liver function test, chest X-ray, HIV, HBsAg, HCV) were within the normal limit except for raised erythrocyte sedimentation rate (ESR) 32mm/h (normal 0–20). Other tumor markers were also within the normal limit: serum lactate dehydrogenase 216 U/L (normal 135–225), serum alpha fetoprotein 1.6 ng/ml (normal 0.89–8.78), serum carcinoembryonic antigen < 0.5 ng/ml (normal 0–5), and serum beta human chorionic gonadotropin < 2 mIU/ml (normal 0–5). Seeing her past history of tuberculosis, interferon gamma release assay was done which was negative. After this, a primary diagnosis of tubo-ovarian abscess was made.

A transabdominal ultrasound (USG)-guided aspiration of the fluid was done and sent for geneXpert®, cell cytology, cell block, and culture (bacterial/fungal/tubercular). GeneXpert® was negative for tuberculosis. The fluid cytology was negative for malignant cells and the cell block showed reactive mesothelial cells and neutrophils. The microbiological reports revealed a sterile bacterial culture. On fungal culture, fungal elements were seen, which were grown on Matrix-assisted laser desorption ionization-time of flight mass spectrometry (MALDI-TOF MS) (Bruker Daltonics, Germany), which showed them to be *Curvularia lunata.*

**Fig. 2:**
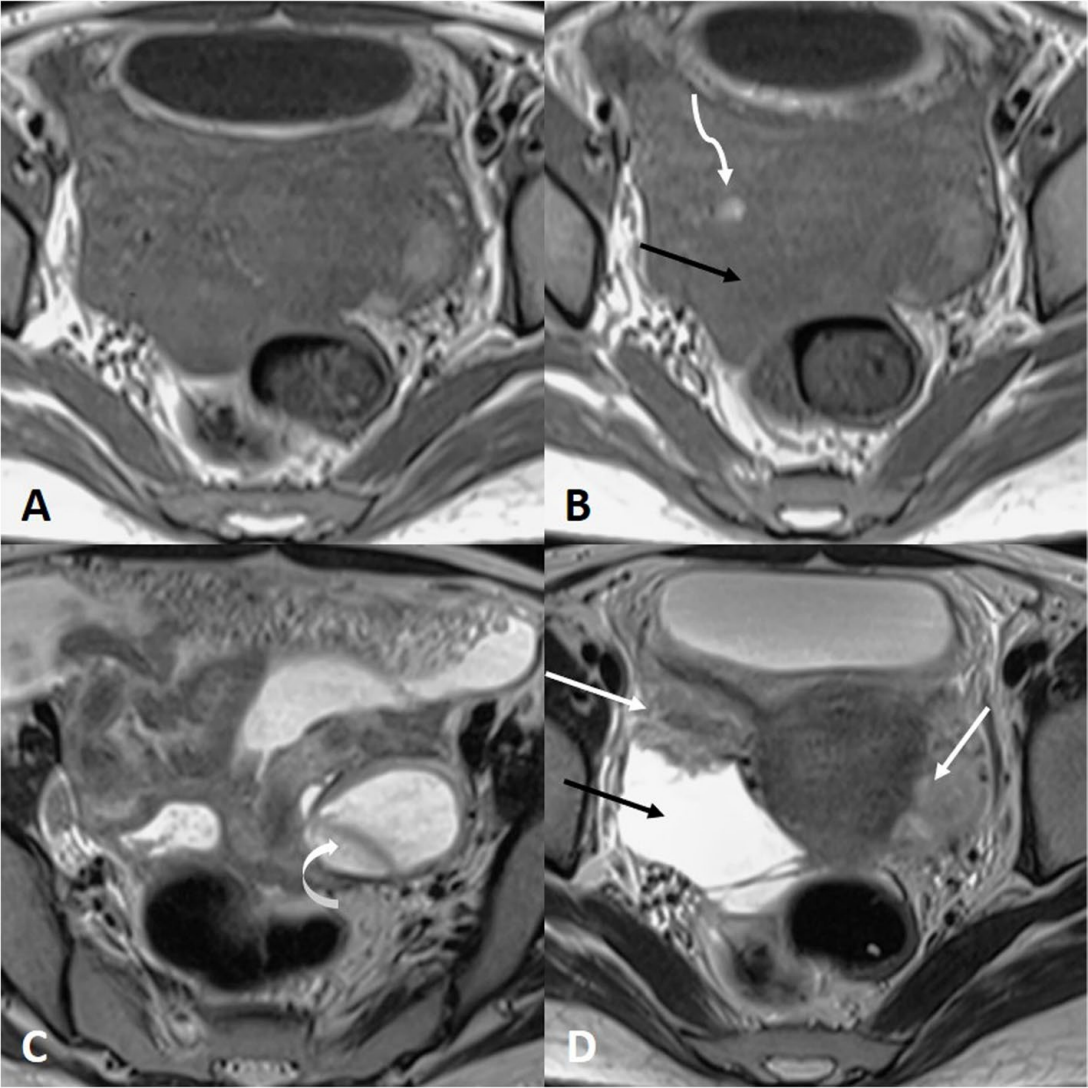
Axial MRI images of the pelvis after treatment showing the uterus in the center with near-total resolution of previously seen mass. Only a small component which has verted to the left side (curved arrow) is remaining. The previously seen calcification is still noted (twisted arrow). The bulk of both ovaries is however visualized normally (straight white arrow). Mild ascites is seen (straight black arrow) which may indicate post-treatment residual peritumoral inflammation

She was started on oral standard itraconazole 200mg once daily and was followed up every week. During the second week of follow-up, an abdominal contrast-enhanced CECT was repeated that showed bilateral adnexal masses with dilated tubular structures with thickened enhancing walls, moderate fluid collection was still seen in the pelvis along with peritoneal thickening, and the lesion also showed calcifications and cystic components of various compositions suggesting a diagnosis of a tubo-ovarian abscess (Fig. 2). So, another therapeutic USG-guided aspiration was done and 80 ml fluid was aspirated.

**Fig. 3:**
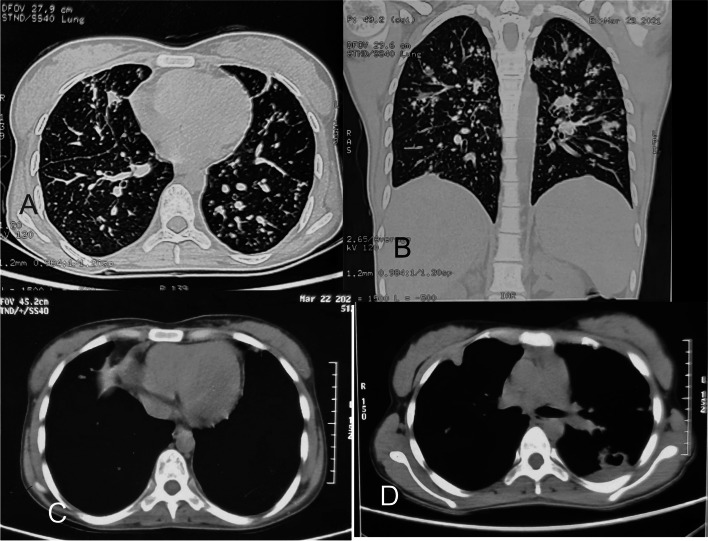
Computerized tomography scan of the lung and thorax showing **A** axial section of bilateral lung showing disseminated tuberculosis lesions in both lungs, **B** sagittal section showing the parenchymal lesions, **C** axial section showing submammary plural collection, and **D** axial section showing subcutaneous collection (cold abscess) at 7 o’clock position

After 6 weeks of itraconazole treatment, she was symptomatically improved. A repeat pelvis MRI was done that showed near-total resolution of mass, and bulk of bilateral ovaries was seen normally (Fig. 2). Again, transabdominal ultrasound-guided fluid aspiration was done from the remaining cyst and sent for repeat fungal culture which was negative for any fungal elements. Itraconazole was stopped and she was kept on regular follow-up. Two months later, the patient presented with a chest wall swelling and weight loss. A CT scan was done that showed miliary tuberculosis and cold abscess in the chest wall (Fig. 3). An aspiration was done and sent for geneXpert® that confirmed MDR tuberculosis; 3 months after the start of antitubercular treatment, the cold abscess was regressed and the patient had reported 3 kg of weight gain.

## Discussion

Fungal peritonitis associated with *Curvularia lunata* is a relatively rare phenomenon. To the best of our knowledge, a total of ten cases of peritonitis caused by the fungal species of *Curvularia* (see supplementary material: Table S1) has been reported in the English literature [1, 3–11] and half of these cases are due to *C. lunata*. All *Curvularia* infections which are reported have been seen only in immunocompromised patients with type 2 diabetes, hypertension, ischemic heart disease, and end-stage renal disease. In this patient, there was a previous history of tuberculosis for which she took ATT and this could be a reason for her compromised immunity. Because of this, she acquired *C. lunata* infection as an opportunistic infection in the presence of her decreased immunity [12] and again she probably had a re-emergence of her latent TB infection, because of her decreased immunity [13]. Tiemesson et al. mentioned that ATT intake can cause defective neutrophil function and affect innate immunity [14]. There is also a possibility that initial investigations of TB might be falsely negative, but since the patient responded to antifungal therapy and there is a resolution of the mass so it goes more towards the fungal infection than tuberculosis.

Curvularia usually spread via inhalational or dermal inoculation routes. All cases which are reported have a dermal inoculation route through a peritoneal dialysis catheter. Three forms of *Curvularia* infection have been observed. They are catheter obstruction without peritonitis, catheter obstruction with peritonitis, and only peritonitis. The present case falls under the last category, i.e., only peritonitis. Since patient’s geneXpert and chest X-ray was normal at presentation, so the possibility of concurrent tuberculosis at initial presentation was ruled out.

TOA is usually a consequence of pelvic inflammatory disease (PID) and most commonly seen in sexually active women of reproductive age. Cho et al. reported a 4.1% incidence of virginal women PID among all cases of PID. Several hypotheses have been proposed for origin in virgin women through the lower genital tract, urinary tract, gastrointestinal tract, and skin wound but none of them was proven [15]. Early diagnosis is the key for management, and ultrasound-guided aspiration and antibiotics are the valid first-line treatment option as we have done in our case. Surgical exploration is only needed in acute cases, if ruptured TOA is suspected [16].

Differentiating these masses on imaging from bacterial or tubercular infection is also important. In genitourinary infections, fungal infections on CT range from homogeneous hypoattenuating masses to heterogeneous masses with areas of hypoenhancement or calcifications. On MR images, the appearance varies according to the acuity of the infection, extent of necrosis, and degree of calcification. Most frequently, the masses are T1 hypointense and mildly T2 hyperintense [17]. Differential diagnosis of abdominal tuberculosis also cannot be ruled out as in wet type it presents primarily either as free or loculated ascites, associated or not with diffuse and smooth peritoneal thickening; in the dry type of presentation, there is predominance of peritoneal and mesenteric thickening with caseous nodules, lymph node enlargement, and fibrinous adhesions; and the fibrous type of presentation is characterized by omental thickening, entanglement of bowel loops, and loculated ascites [18].

The MRI findings in the present case which showed adnexal mass and the raised tumor marker (CA 125) level were initially suggestive of epithelial ovarian malignancy. However, increased level of CA 125 may also be seen in non-malignant conditions such as menstruation, pregnancy, liver disease, congestive heart failure, uterine fibroids, pelvic inflammatory disease, peritonitis, etc. [19] Malignancy and tuberculosis were ruled out from the provisional diagnosis after the cytology, cellblock, and geneXpert® and availability of microbiological reports. Correlation of the clinical features, CT findings, and serological and microbiological studies arrived at the final diagnosis as fungal peritonitis by *Curvularia lunata*. The MALDI-TOF MS library used in the present study offers the most extended but comprehensive technique for the identification of filamentous fungi from clinical samples. Becker et al. compared it with the classical method (microscopy) based on morphological observations. MALDI-TOF correctly identified 95.4% of the total samples using the in-house database, while microscopy identified only 61.5% of the isolates, so MALDI-TOF MS provides reliable identifications and showed a higher accuracy than the traditional approach [20].

In young women, most cases of TOA mimic as pelvic tumors even after imaging studies and they usually undergo a surgical procedure which can easily be avoided and treated by appropriate medical management as the first line of treatment. A radiologist experienced with pelvic imaging is necessary for making or excluding the diagnosis before deciding any plan of management. Of all the *C. lunata* peritonitis cases reported, this is the first case that presented with TOA with peritonitis in a virgin woman.

## Supplementary Information

**Additional file 1: Table S1.** Cases of *Curvularia* peritonitis reported in literature.

## Data Availability

This is a case report; the data is provided in the text of the manuscript.
