# Supplementary material for: Tubo-ovarian mass with raised CA-125 in a 21-year-old female
Source: World J Surg Oncol. 2022 Jun 8;20:188. doi: 10.1186/s12957-022-02651-w (PMC9178888; doi:10.1186/s12957-022-02651-w)
Supplement: Supplementary file 1 — Additional file 1: Table S1. Cases of Curvularia peritonitis reported in literature. [file 12957_2022_2651_MOESM1_ESM.docx]

**Table 1: Cases of *Curvularia* peritonitis reported in literature**

| **Year** | **Author** | **Age/sex** | **Finding** | **Associated disease** | **Organism** | **Therapy** | **Outcome** | |
| --- | --- | --- | --- | --- | --- | --- | --- | --- |
| 1989 | Guarner  et al. | 53/M | Peritoneal dialysis peritonitis | IDDM, hypertension, congestive heart failure and ESRD | C. lunata | 5-Flucytosine, gentamycin and vancomycin | Resolved | |
| 1990 | Ujhelyi et al. | 28/M | PD Peritonitis | Membranoprolifero glomerulonephritis | Curvularia species  Trichosporon | Amphotericin | Resolved | |
| 1994 | Lopes  et al. | 63/M | Peritoneal dialysis peritonitis | IDDM with ESRD | C. lunata | Amphotericin-B | Resolved | |
| 2001 | Canon et al | 11/M | PD Peritonitis | Idiopathic crescentic glomerulonephritis | Curvularia species | Amphotericin-B | Resolved | |
| 2005 | Pimentel et al. | 85/F | PD peritonitis | Renovascular disease | C. inequalis | Amphotericin B | Patient died | |
| 2005 | Vachharajani et al | 45/M | PD peritonitis | ESRD, hypertension, seizure disorder | C. geniculata | Amphotericin B + Oral Itraconazole | Resolved | |
| 2011 | Varughese  et al. | 61/M | Peritoneal dialysis peritonitis | ischemic heart disease, IDDM, hypertension and ESRD | C. lunata | Amphotericin-B | Expired due to septic shock | |
| 2012 | Kalawat  et al. | 55/M | Peritoneal dialysis peritonitis | ESRD nondiabetic | C. lunata | Fluconozale with ceftazidime, cefazolin, amikacin | Resolved | |
| 2013 | Tereda et al. | 61/F | peritonitis | Vulvar cancer, DM | C.geniculata ,Pithomyces | Oral Voriconazole | Not reported | |
| 2016 | Subramanyam et al. | 45/M | Peritoneal dialysis peritonitis | Diabetic nephropathy with ESRD | C. lunata | Oral Voriconazole and Amphotericin-B | | Resolved |
| 2021 | Present Case | 21/F | TOM with fluid collection in pelvis | Past history of tuberculosis | C. lunata | Itraconazole oral | | Resolved |

IDDM-Insulin-dependent diabetes mellitus, ESRD- End stage renal disease, TOM- tuboovarian mass
